# The amino‐terminal tail of Hxt11 confers membrane stability to the Hxt2 sugar transporter and improves xylose fermentation in the presence of acetic acid

**DOI:** 10.1002/bit.26322

**Published:** 2017-05-23

**Authors:** Hyun Yong Shin, Jeroen G. Nijland, Paul P. de Waal, Arnold J. M. Driessen

**Affiliations:** ^1^ Molecular Microbiology, Groningen Biomolecular Sciences and Biotechnology, University of Groningen Zernike Institute for Advanced Materials and Kluyver Centre for Genomics of Industrial Fermentation Nijenborgh 7 9747 AG Groningen The Netherlands; ^2^ DSM Biotechnology Center Delft The Netherlands

**Keywords:** yeast, Hxt2, sugar transport, directed evolution, sugar fermentation, acetic acid

## Abstract

Hxt2 is a glucose repressed, high affinity glucose transporter of the yeast *Saccharomyces cerevisiae* and is subjected to high glucose induced degradation. Hxt11 is a sugar transporter that is stably expressed at the membrane irrespective the sugar concentration. To transfer this property to Hxt2, the N‐terminal tail of Hxt2 was replaced by the corresponding region of Hxt11 yielding a chimeric Hxt11/2 transporter. This resulted in the stable expression of Hxt2 at the membrane and improved the growth on 8% d‐glucose and 4% d‐xylose. Mutation of N361 of Hxt11/2 into threonine reversed the specificity for d‐xylose over d‐glucose with high d‐xylose transport rates. This mutant supported efficient sugar fermentation of both d‐glucose and d‐xylose at industrially relevant sugar concentrations even in the presence of the inhibitor acetic acid which is normally present in lignocellulosic hydrolysates. Biotechnol. Bioeng. 2017;114: 1937–1945. © 2017 The Authors. *Biotechnology and Bioengineering* Published by Wiley Periodicals, Inc.

**Abbreviations**
HXT: hexose transporter
PCR: polymerase chain reaction
XylA: xylose isomerase

## Introduction

Lignocellulosic biomass is an abundant and renewable resource for sustainable bioethanol production that does not compete with food crop (Tilman et al., 2009). However, to utilize this resource for fuel ethanol production, raw material needs to be pretreated. Due to the recalcitrant nature of lignocellulosic materials, harsh conditions have to be used, which result in the production of several by‐products that can have inhibitory effects on microbial metabolism (Klinke et al., 2004). During pretreatment, inhibitors such as furans, phenols, carboxylic and inorganic acids, aldehydes, and alcohols are released from the lignocellulosic biomass and these compounds inhibit xylose fermentation (Demeke et al., 2013; Klinke et al., 2004). For instance, the acetic acid concentration in corn stove and poplar lignocellulosic biomass can be up to 5.6 and 3.6 (w/w)%, respectively (Lu et al., 2009). Acetic acid negatively affects cell growth, the xylose fermentation rate, and ethanol production (Casey et al., 2010; Hasunuma et al., 2011; Sakihama et al., 2015). When the undissociated form of acetic acid enters the cell, the intracellular pH needs to be recovered by pumping out protons at the expense of ATP, which may negatively influence cell growth (Wei et al., 2013). Additionally, engineered yeast strains capable of metabolizing pentose sugars show a reduced ethanol tolerance during xylose fermentation as compared to glucose fermentation (Jeffries and Jin, 2000). As a result, sequential utilization of xylose after glucose depletion is a significant barrier to the full conversion of mixed sugars to ethanol by engineered *Saccharomyces cerevisiae* strains. Therefore, slow xylose conversion rates are a major problem that must be solved to make cellulosic biofuels economically viable using the aforementioned strains.

Despite the fact that yeast does not contain transporter proteins that facilitate specific xylose uptake (Nijland et al., 2014; Young et al., 2014), decades of research have focused on improving the xylose catabolic pathway in recombinant *S. cerevisiae* (Lee et al., 2012, 2014; Runquist et al., 2010a; Wei et al., 2013). Hexose transporters (HXTs) in yeast are efficient glucose transporters and only transport xylose with low affinity. Therefore, previous efforts focused on the identification and introduction of heterologous transporters with a high affinity for xylose (Fonseca et al., 2011; Wang et al., 2013; Weierstall et al., 1999). However, the majority of the heterologous transporters were found to be either nonfunctional, inefficient, instable, or not xylose specific (Hector et al., 2008; Leandro et al., 2006; Runquist et al., 2009, 2010b; Young et al., 2010).

Glucose transport in yeast is mediated by proteins encoded by the *HXT* gene family of which *HXT1‐7* genes have been identified as the metabolically most significant hexose transporters (Kruckeberg, 1996). These *HXT* genes are differentially regulated at the levels of expression and post‐translational inactivation in the response to sugar concentration (Boles and Hollenberg, 1997; Kruckeberg et al., 1999). For instance, yeast expresses the high‐affinity glucose transporter encoding *HXT2* and *HXT7* genes at low‐glucose conditions (Diderich et al., 1999). Importantly, transcription of these genes is repressed at high glucose concentration, while under those conditions, Hxt2 and Hxt7 proteins at the membrane are targeted to the vacuole for degradation (Kruckeberg et al., 1999; Ye et al., 2001). Hxt7 and Hxt2 can be ubiquitinated and phosphorylated at N‐terminal residues (Swaney et al., 2013) and this likely contributes to their degradation.

A possible solution for the xylose transport dilemma is the engineering of endogenous Hxt transporters to make them more specific for xylose transport (Farwick et al., 2014; Young et al., 2014). This can be achieved through mutagenesis of a highly conserved asparagine residue that is part of the sugar binding site. Mutagenesis of this residue alters the glucose over xylose specificity ratio, but a downside of the increased specificity for xylose is that it is usually accompanied with a reduction of the maximal xylose transport rate. By additional mutants, high xylose transport rates can be recovered even exceeding that of glucose (Li et al., 2016). However, such engineered sugar transporters will still be degraded in a glucose concentration dependent manner which makes this method less effective with transporters that are subjected to high glucose induced degradation such as Hxt2 or Hxt7.

Recently, we reported that the cryptic Hxt11 transporter is a low affinity glucose transporter that can be readily engineered into a specific xylose transporter that is stable over a wide range of glucose concentrations (Shin et al., 2015). The Hxt11 mutant is a high capacity xylose transporter but exhibits only a moderate affinity for the pentose sugar. In contrast, Hxt2 shows a much higher affinity for glucose than the native Hxt11 and thus has the potential to function also as a high affinity xylose transporter. A high affinity for xylose would ensure operation at maximal transport rates over a wide range of xylose concentrations during mixed sugar fermentation, and would enable a rapid and more complete depletion of the xylose in the medium. However, Hxt2 is subjected to high glucose induced degradation. It was previously reported that high glucose induced degradation of the Hxt7 transporter is blocked after truncation of its N‐terminal tail, suggesting that this region bear signaling information (Krampe and Boles, 2002). Because of the analogy between Hxt2 and Hxt7, both being subjected to ubiquitination and phosphorylation of N‐terminal residues (Swaney et al., 2013), we hypothesize that replacing the N‐terminal tail of Hxt2 by the corresponding region of Hxt11 will allow stable expression of Hxt2 at the membrane at high glucose concentration. Our data show that the chimeric Hxt11/2 transporter indeed is stably expressed at the plasma membrane under those conditions. By the further introduction of specificity mutations at the conserved asparagine residue of the sugar binding site, a chimeric Hxt11/2 transporter was obtained that supported high capacity xylose transport and even allowed for xylose and glucose co‐metabolism in the presence of a high concentration of the inhibitor acetic acid.

## Materials and Methods

### Yeast Strains and Cultivations

*S. cerevisiae* strains used in this study (Table I) were provided by DSM and may be made available for academic research under a strict Material Transfer Agreement with DSM (contact: paul.waal-de@dsm.com). The culture medium used was a defined mineral medium, with vitamins prepared as described by Verduyn et al. (1992). For storage of the strains, shake flask cultures were performed in the mineral medium supplemented with 2% maltose (Sigma–Aldrich, Rockville, MD) in case of the DS68616 and DS68625‐derivatives, or 2% xylose (Roth) in case of DS71054‐derivatives. With strains DS68625, DS68616, or DS71054, the Verduyn‐urea was supplemented with 0.02% histidine (Sigma–Aldrich) to complement for the auxotrophic marker. Cultures were maintained at 30°C in an orbital shaker until stationary growth phase was reached. After the addition of glycerol to 30% (v/v), samples were stored in 2 mL aliquots at −80°C.

**Table I:** Strains and plasmids

Strain/plasmid	Relevant genotype and/or characteristics	Source or reference
*S. cerevisiae* strains		
DS68616	*Mat a, ura3‐52*, *leu2‐112*, *gre3::loxP*, *loxP‐Ptpi:TAL1*, *loxP‐Ptpi::RKI1*, *loxP‐Ptpi‐TKL1*, *loxP‐Ptpi‐RPE1*, *delta::Padh1XKS1Tcyc1‐LEU2*, *delta::URA3‐Ptpi‐xylA‐Tcyc1*, *delta::his3*	This paper
DS68625	DS68616, *his3::loxP*, *hxt2::loxP‐kanMX‐loxP*, *hxt367::loxP‐hphMX‐loxP*, *hxt145::loxP‐natMX‐loxP*, *gal2::loxP‐zeoMX‐loxP*	This paper
DS71054	DS71055, *glk1::lox72; hxk1::loxP; hxk2::lox*72; *gal1::lox*P; *his3*::loxPnatMXloxP	This paper
DS71055	DS68616‐derivative after evolutionary engineering	DSM, the Netherlands
Plasmids		
pRS313	*E. coli*/yeast shuttle vector; *CEN6*, *ARSH4*, *HIS3*, Amp^r^	Sikorski and Hieter (1989)

### Genomic DNA Isolation

Genomic DNA was isolated after 16 h of growth of the cells in the mineral medium containing 2% maltose using a modified yeast genomic DNA isolation protocol (Harju et al., 2004). Yeast pellets from 2 mL of exponential phase cell culture were mixed with 0.2 mL of glass beads (diameter, 0.45 mm) and disrupted in a Fastprep FP120 (Bio‐101, Thermo Savant, CA) by a 45 s burst at speed 6.

### Overexpression *Chimeric HXT11/2* and *HXT2* in *S. cerevisiae*

For PCR amplification, Phusion® High‐Fidelity PCR Master Mix with HF buffer was used (Finnzymes; Fisher Scientific, Landsmeer, the Netherlands). Restriction enzymes and T4 DNA ligase were acquired from Fermentas (Fisher Scientific). The open‐reading frames for *HXT11* and *HXT2* were PCR‐amplified from gDNA of strain DS68616 using primers F HXT11/R HXT11, and F HXT2/R HXT2 (Supplementary Table SII). To construct of *chimeric HXT11/2*, 1st loop of HXT11 and HXT2 was PCR‐amplified using F HXT11/R HXT2 TM1, and F HXT2 TM1/R HXT2. And the fragments were used as template DNA of *chimeric HXT11/2* in an overlap PCR using the outside primers F HXT11 and R HXT2 and cloned into pRS313P7T7 using XbaI and Cfr9I.

The HXT11 and HXT2 PCR fragments were sequenced for validation, cut using restriction enzymes XbaI and Cfr9I (Fermentas) and cloned in yeast expression vector pRS313‐P7T7 behind the constitutive HXT7 (−391) promoter (P7) preceding the HXT7 terminator (T7). Plasmids were amplified and maintained in *Escherichia coli* DH5α cells. Plasmids were isolated from *E. coli* cultures using the GeneElute plasmid Miniprep kit (Sigma–Aldrich). The chimeric *HXT11/2* and *HXT2* expression construct were transformed into *S. cerevisiae* DS68625 using standard yeast genetic techniques (Gietz and Woods, 2006).

### Construction of Chimeric *HXT11/2‐GFP* and *HXT2‐GFP* Expression Plasmids

The GFP gene in plasmid pDONR‐eGFP‐AT (Kovalchuk et al., 2012) was amplified by PCR with the primers GFP‐F and GFP‐R, and the product was cloned into the XmaI and ClaI restriction sites of pRS313‐P7T7. The resulting plasmid and PCR products of the *HXT2* and *chimeric HXT11/2* were digested with XbaI and Cfr9I, and ligated with the GFP cassette. The chimeric *HXT11/2‐GFP* and *HXT2‐GFP* genes were validated by DNA sequencing.

### Mutagenesis and Screening for Xylose Transport Mutants of Chimeric Hxt11/2

Saturated mutagenesis of position N361 in Hxt2 was done by PCR using Phusion® High‐Fidelity PCR Master Mix with HF buffer using primer pairs F HXT11/R HXT2 361NNN and F HXT2 361NNN/R HXT2 (Supplementary Table SIII). The fragments of 1,103 and 542 base pairs were subsequently used in an overlap PCR using the outside primers F HXT2 and R HXT2 and cloned into pRS313P7T7 using XbaI and Cfr9I. Sequencing of 48 *E. coli* clones yielded N361S (agt), N361P (ccc), N361G (ggg), N361A (gcg), N361R (cgg), N361F (ttt), N361T (acg), N361K (aaa), N361Q (caa), N361I (att), and N361V (gtg). The remaining eight amino acids at position 361 were amplified and cloned as mentioned above with overlap PCR using specific primers in which the NNN was replaced by ctt (L), tat (Y), cat (H), atg (M), gat (D), gaa (E), tgt (C), and N361W (tgg).

The library of fragments were cloned into pRS313‐P7T7 vector and transformed into the strain DS71054 that is unable to grow on glucose but that contains the xylose metabolic pathway. All chimeric Hxt11/2 variants were screened by growing transformants on a 1:5 ratio of xylose (1%) and glucose (5%) with mineral medium using the 96‐well plate format and Synergy MX (Bio Tek Instruments, Inc. VT, US) reader.

### Uptake Measurements

The uptake of radiolabeled xylose or glucose was measured as follows: cells were collected by centrifugation (3,000 rpm, 3 min, 20°C), washed and suspend into mineral medium. [^14^C] xylose or [^14^C] glucose (CAMPRO Scientific GmbH, Veenendaal, the Netherlands) stocks were added to the cells, uptake reactions were stopped at various time intervals by addition of 5 mL of 0.1 M lithium chloride, and the suspension was filtered (0.45 µm HV membrane filter, Milipore, France). Filters were washed with another 5 mL of lithium chloride and counted with the emulsifier scintillator plus (Perkin‐Elmer). Uptake experiments with strain DS68625 expressing Hxt11‐variants were done with 0.5–500 mM xylose or 0.1–500 mM glucose. In general, the uptake of xylose was monitored after 1 min and for glucose after 15 s in quadruple. Glucose competition studies were performed with 50 mM [^14^C] xylose in the presence of 50–500 mM unlabeled glucose.

### Fermentation

Yeast cultures were grown in mineral medium containing 2% maltose to prepare inoculums for glucose and xylose fermentation experiments. Cells at mid‐exponential phase were harvested and inoculated after wishing twice by sterilized water. Fermentation experiments were performed at 30°C using 200 mL of mineral medium containing 8% glucose and 4% xylose in 250 mL bottle under oxygen limited conditions. The initial OD_600_ was 2. All bottle‐based fermentation experiments were repeated independently. When indicated, the medium was supplemented with 4 g/L acetic acid, and the pH was adjusted to 5.5 using KOH.

### Fluorescence Microscopy

Plasmid pRS313‐P7T7:*GFP‐HXT11/2* and *GFP‐HXT/2* was transformed into *S. cerevisiae* strain DS68625. Individual colonies were used to inoculate minimal medium containing 2% glucose, and cells recovered from the exponential growth phase (at an optical density of 10 at 600 nm) were subjected to fluorescence microscopy using a Nikon Eclipse‐Ti microscope equipped with a 100× oil immersion objective, a filter set for GFP, and a Nikon DS‐5Mc cooled camera (Nikon, Japan). Routinely, at least 100 cells per sample were examined, and each experiment was replicated at least three times.

### Miscellaneous Analytical Methods

*S. cerevisiae* cells were grown in the mineral medium supplemented glucose and xylose with the initial pH adjusted to 5.5. Cell growth was monitored by optical density (OD) at 600 nm using UV–visible spectrophotometer. The concentration of glucose, xylose, and ethanol was measured by HPLC (Shimadzu, Kyoto, Japan) using an Aminex HPX‐87H column (Bio‐Rad) and a refractive index detector (Shimadzu, Kyoto, Japan). The temperature of the column and detector was maintained at 65°C. The mobile phase was 0.005 N H_2_SO_4_ at a flow rate of 0.55 mL/min.

## Results and Discussion

**Expression of Chimeric HXT11/2 Genes in *S. cerevisiae* Strain DS68625**

**Figure 1:**
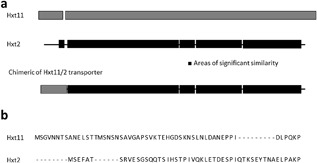
Scheme of the chimeric Hxt11/2 sugar transporter of *S. cerevisiae*. (**a**) The Hxt11/2 chimera was derived from HXT2 in which the N‐terminal, cytosolic part of the HXT2 coding sequence was replaced by the corresponding region of HXT11. (**b**) Aligment of the amino acid sequence of the cytosolic N‐terminus of Hxt2 and Hxt11.

**Figure 2:**
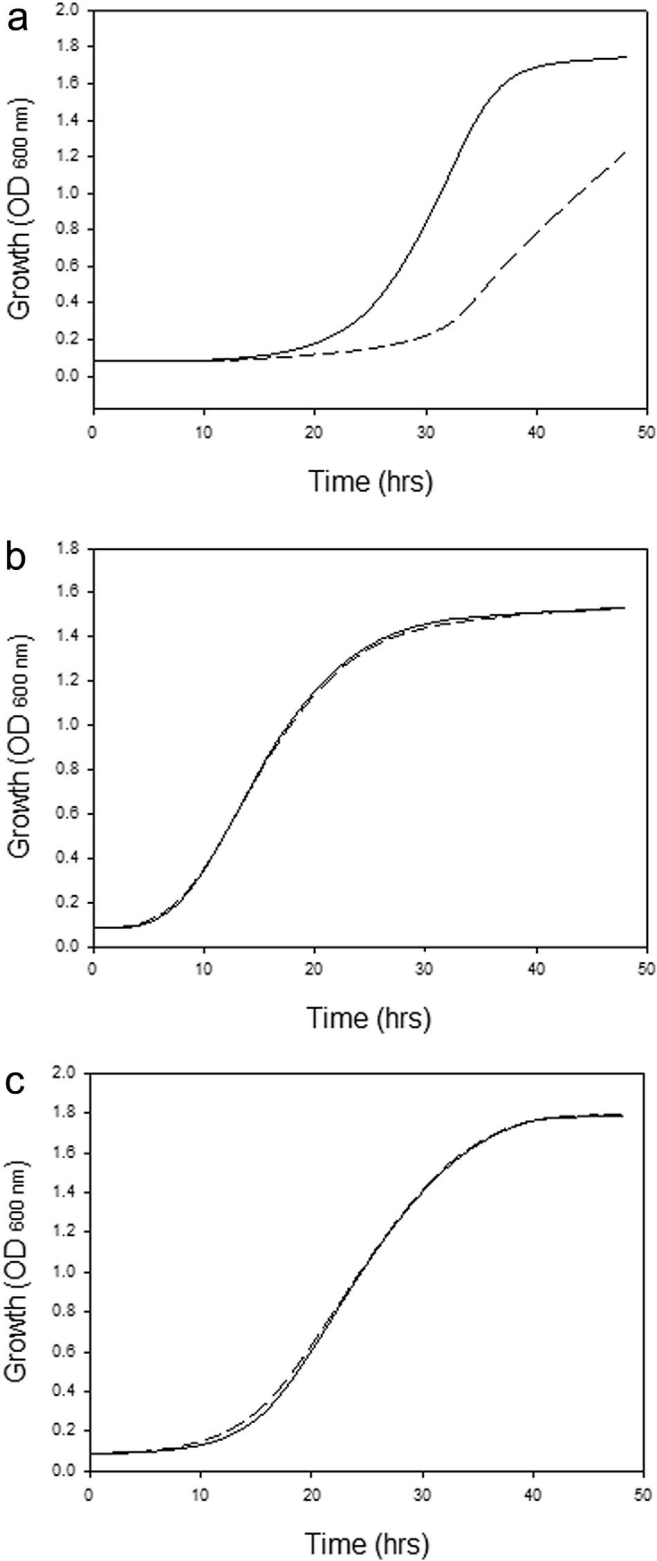
Growth of *S. cereviseae* strain DS68625 expressing wild‐type Hxt2 hexose transporter (‐‐) and the chimeric Hxt11/2 hexose transporter (**−**) on (**a**) 6% d‐glucose. (**b**) 2% d‐xylose, and (**c**) 2% d‐glucose.

Hxt1‐7 sugar transporters are subjected to degradation in response to the glucose level in the medium (Ozcan and Johnston, 1999). The recently described Hxt11 transporter, however, is stably expressed at the membrane irrespective of the sugar concentration (Shin et al., 2015). Hxt2 is a high capacity glucose transporter that exhibits a high affinity for glucose but that is degraded at high glucose concentrations (Kruckeberg et al., 1999). The exact mechanism of high glucose induced degradation of Hxt2 is not known, but for several other Hxt transporters that are subjected to degradation at low glucose concentration, signaling involves ubiquitination of lysine residues at the N‐terminal tail (Roy et al., 2014). Therefore, we hypothesized that when the N‐terminus of Hxt2 is replaced for the corresponding region of Hxt11, stable membrane expression of Hxt2 might be obtained even at high glucose concentration when the gene is expressed under control of the constitutive truncated HXT7 promoter that was also used in previous studies (Shin et al., 2015). The N‐terminal region of Hxt2 and Hxt11 is poorly conserved (Fig. 1b) in contrast to the overall sequence homology between both transporters which is about 21% (Fig. 1a). Residues 1–49 of Hxt2 were replaced by residues 1–54 of Hxt11, yielding a chimeric Hxt11/2 transporter (Fig. 1). The resulting chimer was expressed in the xylose metabolizing *S. cereviseae* strain DS68625 (Shin et al., 2015) (Table I) that lacks the *HXT1‐7* and *GAL2* genes and, therefore, is unable to grow on glucose or xylose. Cells expressing the wild‐type *HXT2* and chimeric *HXT11/2* genes were grown on high (6%) glucose medium. Growth of the strain expressing the chimeric Hxt11/2 was faster than of cells expressing Hxt2 due to improved sugar consumption (Fig. 2a). Growth of the various strains was similar on low glucose media, that is, media containing either only 2% glucose or 2% xylose (Fig. 2b and c). These data demonstrate the replacement of the N‐tail of Hxt2 for the corresponding region of Hxt11 results in improved growth at high glucose concentration.

Hxt3 is expressed at high glucose concentration but disappears from the membrane at low glucose concentration (Ozcan and Johnston, 1999) via ubiquitination (Snowdon and Merwe, 2012). Recently, we demonstrate that substitution of the N‐terminal lysine residues of the endogenous, but fused hexose transporters Hxt36 resulted in improved membrane localization and cell growth on xylose up to the late stage of sugar fermentation (Nijland et al., 2016). Ubiquitylation plays a prominent role in hexose transporters degradation (Horak and Wolf, 1997; Nijland et al., 2016; Roy et al., 2014), whereas phosphorylation is the primary mechanism for regulating cellular signaling (Humphrey et al., 2015). However, distinct phosphorylation sites are often used in conjunction with ubiquitylation in degradation, and these distinct sites are more highly conserved than the entire set of phosphorylation sites (Swaney et al., 2013). Hxt2 can be ubiquitinated at residue K26 and this triggers its inactivation. Furthermore, Hxt2 contains a series of serine and threonine residues at its N‐terminal domain that can be phosphorylated (http://www.yeastgenome.org/locus/S000004613/protein). Although the N‐terminal region of Hxt11 also contains potential ubiquitination and/or phosphorylation sites (http://www.yeastgenome.org/locus/S000005516/protein), but this region is poorly conserved with Hxt2 (Fig. 1) while it is unknown if a similar type of modification occurs. Rather, we previously observed stable Hxt11 membrane expression over a wide range of glucose concentrations (Shin et al., 2015). Possible a lower propensity for modification of this region may render the chimeric Hxt11/2 more stable at the membrane at high sugar concentrations.

### Chimeric Hxt11/2 Is Localized at the Membrane at High Glucose Concentration

**Figure 3:**
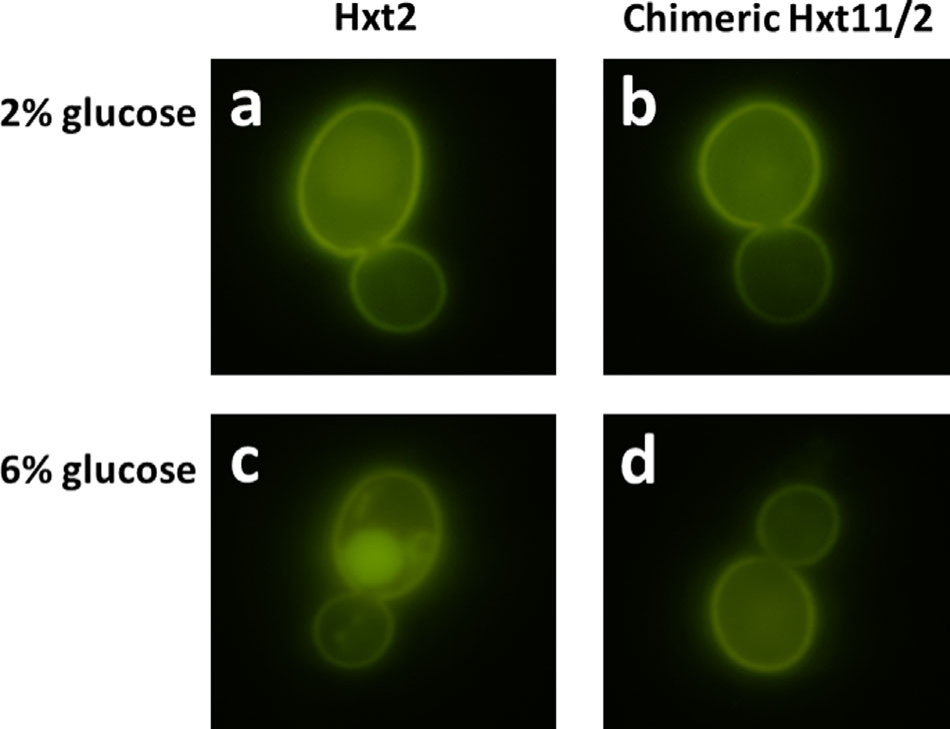
Fluorescence microscopy imaging of the cellular localization of GFP‐fused Hxt transporters expressed in *S. cereviseae* DS68625. Membrane localization of (**a**) HXT2‐GFP and (**b**) chimeric HXT2‐GFP on 2% glucose, and of (**c**) HXT2‐GFP, and (**d**) chimeric HXT2‐GFP on 6% glucose. Cells were incubated with the indicated sugars for 2 h before microscopy.

To examine the expression and cellular localization of Hxt2 and the chimeric Hxt11/2, a C‐terminal fusion to GFP was constructed that was examined by fluorescence microscope. When expressed in strain DS68625 that was grown on 2% glucose, both Hxt2 and the chimeric Hxt11/2 fusion proteins predominantly localized to the cell membrane (Fig. 3a and b). However, when cells were grown with 6% glucose, most of the Hxt2‐GFP fusion localized to the vacuole likely for degradation (Fig. 3c). In contrast, the chimeric Hxt11/2‐GFP fusion remained plasma membrane localized with little fluorescence in the vacuole (Fig. 3d). These data show that stable membrane localization of Hxt2 can be obtained at high glucose concentration when the N‐terminal tail of Hxt2 is replaced for the corresponding region of Hxt11. It thus seems that this region of Hxt2 plays a critical role in its glucose induced degradation. In this respect, of the 20 members of the *HXT* gene family, Hxt2, Gal2, or Hxt7 are sufficient for growth on low glucose concentrations, consistent with their high‐affinity for glucose. These three transporters are all inactivated and degraded at high glucose concentration (Ozcan and Johnston, 1999). Although the exact mechanism of glucose‐induced inactivation is unknown, the process involves internalization of the transporter by endocytosis and subsequent degradation in the vacuole (Busturia and Lagunas, 1986; Horak and Wolf, 1997; Krampe et al., 1998; Kruckeberg et al., 1999). Interestingly, internalization and degradation of Hxt7 was blocked after truncation of its N‐terminal hydrophilic domain (Krampe and Boles, 2002), but the truncation also caused a major loss in activity. Furthermore, when endocytosis was genetically blocked, GFP tagged Hxt7 and Hxt2 remained plasma membrane localized even at high glucose concentration (Krampe and Boles, 2002; Kruckeberg et al., 1999). The recently characterized Hxt11 transporter appears invariant to high glucose‐induced degradation (Shin et al., 2015), and our data now suggest that this property can be transferred to Hxt2 by substituting its N‐terminus for the N‐tail of Hxt11.

### Engineering of Chimeric Hxt11/2 Variants for Improved Xylose Transport

**Figure 4:**
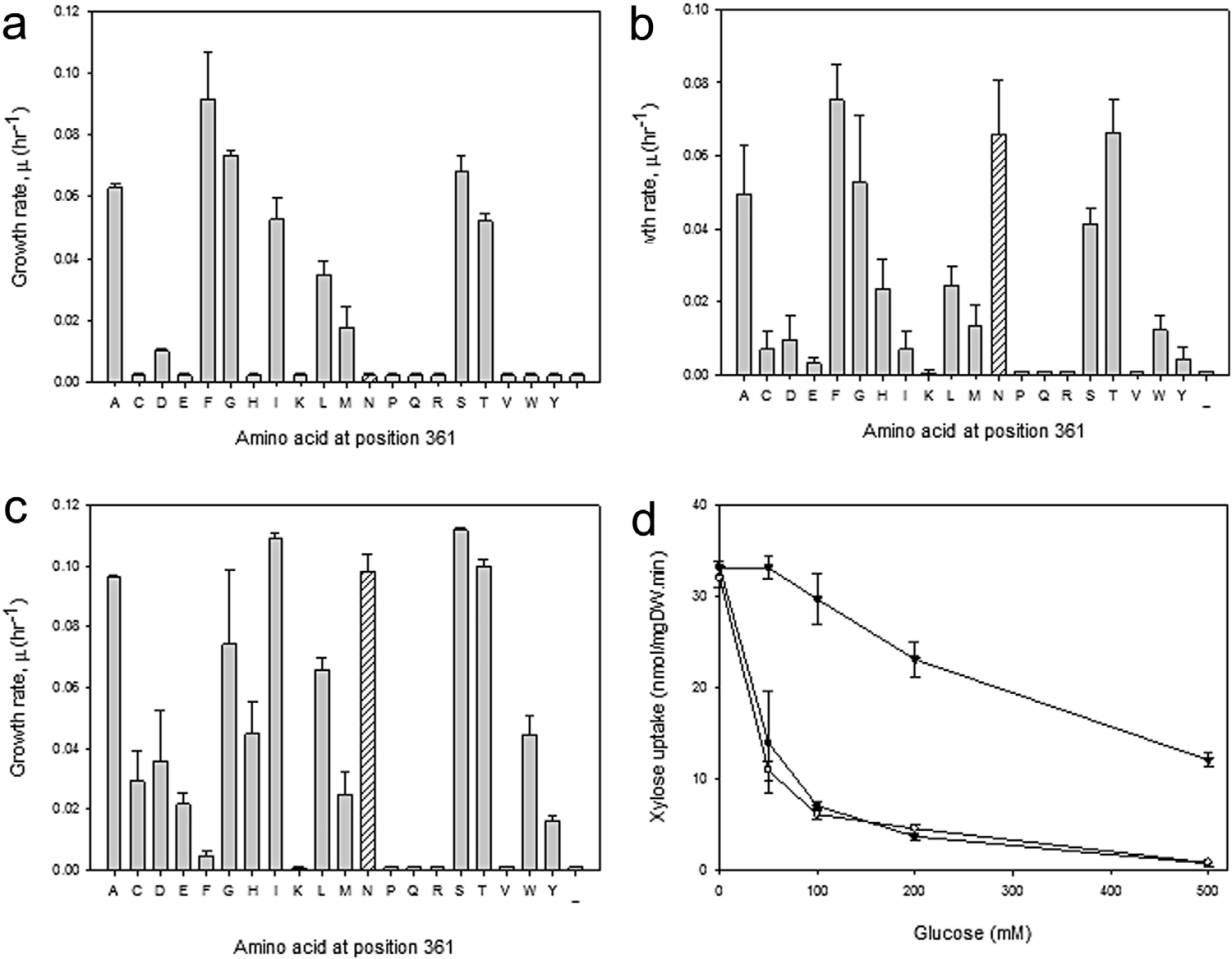
Characterization of the growth of strain DS71054 expressing N361X mutants of the chimeric *HXT11/2* gene. (**a**) Exponential growth rates (*μ*) of strain DS71054 expressing the indicated chimeric Hxt11/2‐N361X mutants when grown on 5% glucose and 1% xylose. The dashed line indicates the growth rate of strain DS71054 without any introduced transporter. (**b**) Exponential growth rates (*μ*) of strain DS68625 expressing the indicated chimeric Hxt11/2‐N361X mutants when grown on 2% xylose. (**c**) Exponential growth rates (*μ*) of strain DS68625 expressing the indicated chimeric Hxt11/2‐N361X mutants when grown on 2% glucose. (**d**) Uptake of [^14^C‐] xylose by the DS68625 strain expressing Hxt2 (○), chimeric Hxt11/2 (•), and Hxt11/2‐N361T (▴) in the presence of increasing concentrations of glucose. The error bars represent the standard error of the mean from two technical samples. The dashed bar indicated the wild‐type activity.

Previous studies demonstrated that the conserved asparagine at position 366 of Hxt11 is a key determinant for the sugar specificity (Nijland et al., 2014; Shin et al., 2015). To improve on the xylose transport specificity of the chimeric Hxt11/2 transporter, the *HXT2* gene was mutagenized at the corresponding position N361. Herein, N361 was replaced with each of the other 19 amino acids to generate a series of N361X mutants using specific primers (Supplementary Table SIV). Mutagenized chimeric *HXT11/2* genes were cloned into the yeast vector pRS313‐T7P7 and transformed into *S. cereviseae* strain DS71054 (Shin et al., 2015). This strain is derived from the xylose metabolizing *S. cerevisiae* strain DS71055, and lacks the four genes with glucose kinase activity, that is, *GLK1*, *HXK1*, *HXK2*, and *GAL1* (Shin et al., 2015). The glucose kinase deficient strain is unable to grow on glucose, but grows on xylose as it contains a xylose metabolic pathway based on the xylose isomerase *xylA* gene. The clones were screened for growth on xylose (1%) in the presence of a fivefold excess of glucose using a 96‐well plate format. This screen yielded six mutants that showed faster growth than the strain containing the original chimeric Hxt11/2 transporter. These six mutations are N361F, N361A, N361I, N361G, N361S, and N361T. Mutants were also tested for growth on glucose when expressed in the transporter‐deficient strain DS68625. The N361S, N361T, N361I, and N361A mutations supported improved growth on glucose as compared to the chimeric Hxt11/2 strain (Fig. 4c). The N361F mutant showed impaired growth on glucose compared to the chimeric Hxt11/2 (Fig. 4c).

Because of the marked effects of the N361 mutations in the chimeric Hxt11/2 transporter on the sugar specificity, these mutants were further examined in the transporter‐deficient strain DS68625 for their ability to support sugar fermentation on 80 g/L d‐glucose and 40 g/L d‐xylose under anaerobic conditions (Supplementary Fig. S1). The chimeric Hxt11/2 N361F mutant (Supplementary Fig. S1h) supported a near to perfect co‐consumption of glucose and xylose, except for the early stages of fermentation at very high glucose concentrations where xylose metabolism lags behind. As compared to the chimeric Hxt11/2 transporter, the overall fermentation by cells expressing the chimeric Hxt11/2 N361T mutant is improved due to an increased rate of xylose consumption (Supplementary Fig. S1b and g). This is accompanied with reduced glucose consumption.

### Transport Characteristics of Chimeric Hxt11/2 Transporters

To gain further insight in the performance of the chimeric Hxt11/2 N361T mutant, xylose transport studies were performed in the presence of increasing concentrations of glucose. Xylose uptake by the chimeric Hxt11/2 N361T mutant expressed in strain DS68625 was strikingly less sensitive to glucose inhibition than wild‐type Hxt2 or the chimeric Hxt11/2 transporter (Fig. 4). Next, the kinetic properties of glucose and xylose uptake by these transporters were determined. Replacement of the N‐tail of Hxt2 for the corresponding region of Hxt11 did not affect the *K*_m_ and *V*_max_ for glucose and xylose uptake showing similar kinetics as the wild‐type Hxt2. The *K*_m_ value for glucose uptake by the chimeric Hxt11/2‐N361T mutant, however, was increased 6.4‐fold, while the *K*_m_ for xylose uptake was improved 1.2‐fold (Table II). Interestingly, the *V*_max_ for glucose and xylose uptake was slightly increased as compared to the wild‐type Hxt2 and chimeric Hxt11/2 transporters. These data demonstrate that the N361T mutation in the chimeric Hxt11/2 renders this transporter more specific for xylose than for glucose, while still maintaining a high transport rate.

**Table II:** *K*_m_ and *V*_max_ values for d‐glucose and d‐xylose uptake by Hxt2 transporters expressed in strain DS68625

	*K*_m_ (mM)			*V*_max_ (nmol/mg DW.min)		
Transporter	Glucose	Xylose	*K*_m_ Glc/Xyl (ratio)	Glucose	Xylose	Source
Hxt2	18.9 ± 2.6	65.5 ± 7.0	0.29	94.5 ± 1.2	42.8 ± 5.2	This study
Chimeric Hxt11/2	17.4 ± 3.8	69.0 ± 9.1	0.25	91.9 ± 6.2	39.8 ± 5.6	This study
Chimeric Hxt11/2‐N361T	107.5 ± 6.6	57.3 ± 5.2	1.88	131.8 ± 5.0	45.6 ± 0.4	This study
Hxt11	33.4 ± 1.2	84.2 ± 10.0	0.40	156.4 ± 7.6	84.6 ± 2.4	Shin et al. (2015)
Hxt11‐N366T	194.4 ± 47.9	46.7 ± 2.7	4.16	238.6 ± 7.4	76.2 ± 4.8	Shin et al. (2015)
Gal2	1.5 ± 0.2	225.6 ± 15.8	0.01	27.2 ± 0.9	91.3 ± 3.2	Farwick et al. (2014)
Gal2‐N376V	22.7 ± 0.2	168.3 ± 31.6	0.16	50.5 ± 1.4	28.4 ± 2.3	Farwick et al. (2014)
Hxt7	0.5 ± 0.1	200.3 ± 13.2	0	26.0 ± 1.1	67.0 ± 2.0	Farwick et al. (2014)
Hxt7‐N370S	10.8 ± 1.0	169.9 ± 26.3	0.06	47.3 ± 1.2	24.1 ± 1.6	Farwick et al. (2014)

n.d., not determined; ±, standard deviation with *n* = 2.

Current transporter engineering is focused on obtaining a specific xylose transporter that is no longer inhibited by glucose. Hence, earlier studies were focused on re‐programming the yeast endogenous sugar transporters for effective xylose transport (Farwick et al., 2014; Nijland et al., 2014; Shin et al., 2015; Young et al., 2012, 2014). In this respect, the Gal2‐N376V and Hxt7‐N370S mutants showed impaired glucose transport with a slight improvement in the affinity for xylose transport. However, with these mutations, an up to threefold drop in the *V*_max_ for xylose uptake occurred compared to the wild‐type Gal2 and Hxt7 proteins (Table II) making them unsuitable for co‐fermentation trials. We have previously shown that with the Hxt11 N366T and N366M mutants, effective sugar co‐metabolism can be realized as these mutations cause only a minor loss in *V*_max_ for xylose uptake (Table II). In contrast, the N361T mutation in the chimeric Hxt11/2 transporter improved both the *K*_m_ and *V*_max_ for xylose uptake (Table II), while for glucose, high transport rates were maintained despite a reduced affinity thus making the chimer an excellent candidate for sugar co‐fermentation.

### Fermentation of Glucose and Xylose in the Presence of Acetic Acid

**Figure 5:**
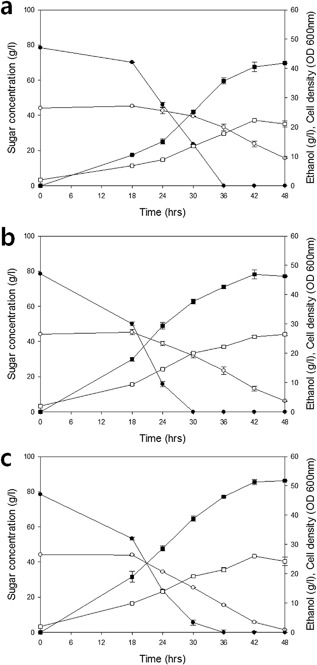
Mixed sugar fermentation in the presence of 4 g/L of acetic acid by strain DS68625 expressing different Hxt2 variants. Consumption of xylose and glucose by the strain DS68625 expressing (**a**) Hxt2, (**b**) chimeric Hxt11/2, or (**c**) chimeric Hxt11/2‐N361T transporter. Symbols used: glucose (•), xylose (○), ethanol (▪), and cell density (□). The initial xylose and glucose concentration was 4% and 8%, respectively. The error bars are the standard error of the mean from three independent experiments with triple samples.

A current challenge of the cellulosic ethanol industry is to counteract the inhibitory effect of acetic acid present in biomass hydrolysates. Glucose‐ and xylose‐specific consumption rates decrease with the concentration of acetic acid, but the inhibitory effect is more severe for xylose consumption as compared to glucose consumption (Casey et al., 2010). Thus, the presence of acetic acid in fermentation media leads to a significant decrease in the observed maximum cell biomass concentration during xylose consumption. Therefore, in addition to the mixed sugar fermentation (Supplemental Fig. S1) we also assessed mixed sugar fermentation in the presence of a high acetic acid (4 g/l) concentration. In contrast to the wild‐type Hxt2 (Fig. 5a) and chimeric Hxt11/2 (Fig. 5b) transporters, the chimeric Hxt11/2‐N361T mutant maintained the ability to co‐metabolize glucose and xylose even in the presence of 4 g/L acetic acid at pH 5.5 (Fig. 5c). The mutant also showed a higher rate of xylose utilization and ethanol productivity as compared to Hxt2 and the original chimeric Hxt11/2 transporter (Fig. 5). Wild‐type Hxt2 (Fig. 5a) showed a severely delayed consumption of the sugars (Fig. 5b and c) likely because of its degradation at high glucose concentrations.

Under sugar co‐metabolizing conditions, the specific xylose consumption rate in the presence of acetic acid with the chimeric Hxt11/2 N361T mutant was 0.38 g/g cell · h which compares favorable to the values of 0.24 and 0.28 g/g cell · h for Hxt2 and the chimeric Hxt11/2 transporter, respectively. In addition, the ethanol productivity of the mutant of chimeric Hxt11/2 transporter was increased to 1.17 g/L · h compared to 1.08 g/L · h and 0.96 g/L · h for the chimeric Hxt11/2 transporter and wild‐type of Hxt2 (Supplementary Table SI). Taken together these results demonstrate that the chimeric Hxt11/2 N361T transporter allows for effective sugars metabolism even in the presence of high concentrations of acetic acid.

Recently, improved co‐fermentation of glucose and xylose was reported upon evolution engineering, and this was accompanied with a slightly increased expression of Hxt2 (Vilela et al., 2015). However, the observed xylose fermentation in the presence of glucose showed a complex pattern with an initial rapid phase of metabolism that leveled off, followed by a late phase of xylose consumption when all glucose was depleted. This complex fermentation pattern suggests the involvement of multiple transporters in the phenotype. Furthermore, the evolved strain was not subjected to genome sequencing, and thus various mutations may have contributed to the characteristics of the strain.

Here, we have engineered the high affinity sugar transporter Hxt2 by stabilizing the protein at the membrane employing the N‐tail of Hxt11. Interestingly, we observed that the resulting chimeric Hxt11/2 transporter with the xylose specificity mutation N361T supports better cell growth, xylose consumption, and ethanol yields on a mixture of glucose and xylose in the presence of acetic acid as compared to the native Hxt2 and the chimeric Hxt11/2 transporter without this mutation (Fig. 5).

## Conclusions

Stable membrane expression of the high affinity glucose transporter Hxt2 at the membrane at high glucose concentrations can be realized by replacing the N‐terminus of Hxt2 for the corresponding region of Hxt11. By the additional introduction of a xylose specificity conferring mutation, a chimeric Hxt11/2‐N361T transporter is obtained that supports effective xylose uptake even at high glucose concentrations in the presence of acetic acid, and that is no longer degraded under those conditions. Therefore, transporter stability and balanced mixed sugar uptake are essentially elements to be taken into account when developing an efficient industrial sugar utilization platform.

## Authors’ Contributions

HS, JN, PW, and AD conceived and designed the research; HS performed the molecular biology and fermentation experiments and drafted the manuscript. JN participated in the design of the transporter mutant library. PW constructed hexokinase deletion strains; PW and AD supervised the project; the manuscript was written by contributions of all authors. All authors read and approved the final manuscript.

The research has been financially supported by the research program of the biobased ecologically balanced sustainable industrial chemistry (BE‐BASIC).

## Supporting information

Additional supporting information may be found in the online version of this article at the publisher's web‐site.

**Supporting Data S1.**
