# Supplementary material for: The amino‐terminal tail of Hxt11 confers membrane stability to the Hxt2 sugar transporter and improves xylose fermentation in the presence of acetic acid
Source: Biotechnol Bioeng. 2017 May 23;114(9):1937–45. doi: 10.1002/bit.26322 (PMC5575463; doi:10.1002/bit.26322)
Supplement: Supplementary file 1 — Supporting Data S1. [file BIT-114-1937-s001.docx]

**
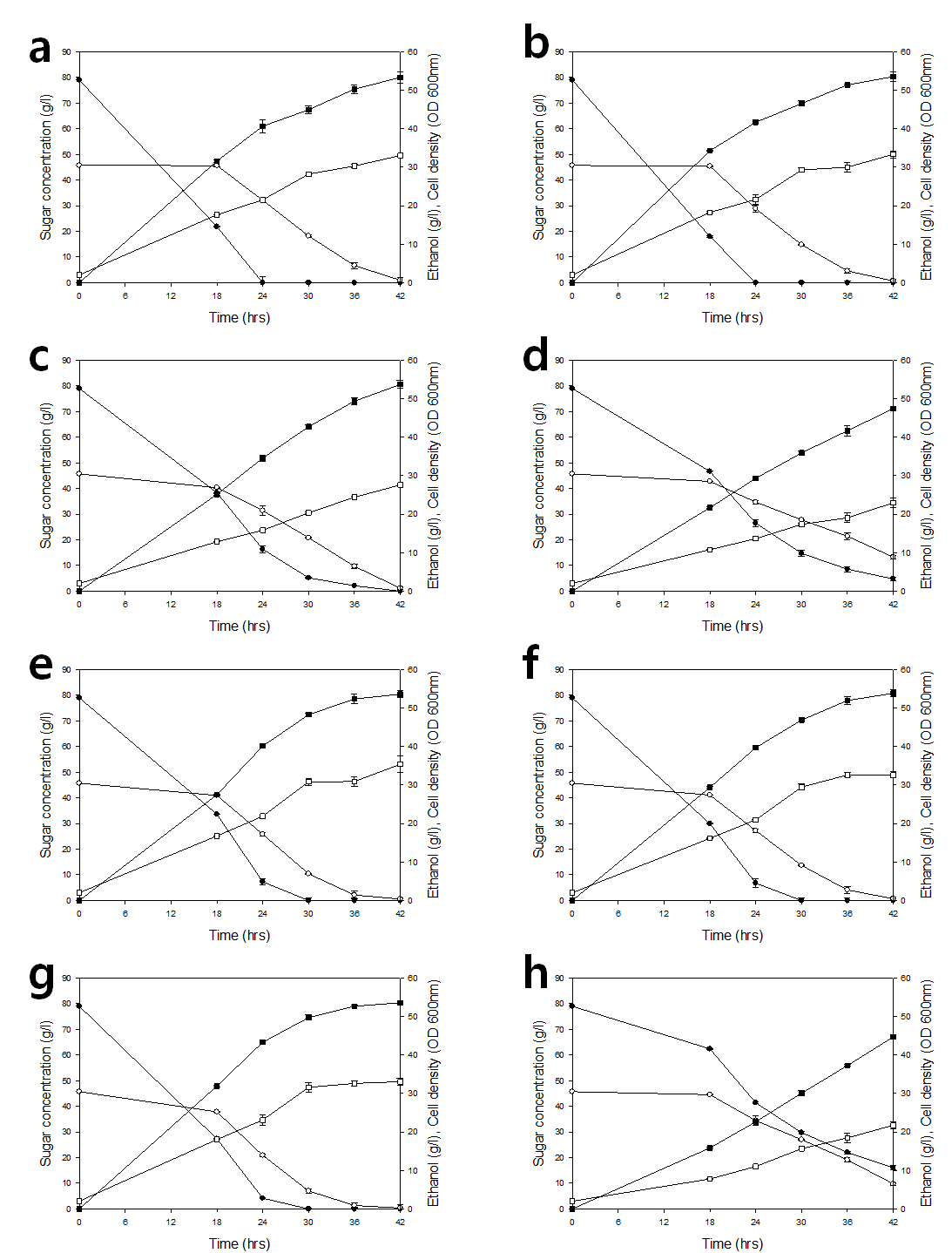
**

**Supplementary Fig. 1.** Consumption of 4% xylose and 8% glucose by the transporter-deficient strain DS68625 expressing Hxt2 (a), or the chimeric Hxt11/2 (b), Hxt11/2-N361A (c), Hxt11/2-N361I (d), Hxt11/2-N361G (e), Hxt11/2-N361S (f), Hxt11/2-N361T (g), and Hxt11/2-N361F (h). Symbols: glucose (●), xylose (○), biomass (□)and ethanol (■). The error bars represent the standard error of the mean from two technical samples

**Supplementary Table 1.**

**Fermentation profiles by the selected *S. cerevisiae* strain expressing Hxt2, the chimeric Hxt11/2 and chimeric Hxt11/2 N361T mutants during anaerobic batch cultivation in the presence of acetic acid.**

|  | DS68625 | | |
| --- | --- | --- | --- |
|  | Hxt2 | Hxt11/2 | Hxt11/2-N361T |
| Q glucose (g glucose/g cell·h)  Q xylose (g xylose/g cell·h)  Q ethanol (g ethanol/g cell·h)  P ethanol (g ethanol/l·h)  Y ethanol (g ethanol/g sugars) | 1.58 ± 0.42  0.24 ± 0.12  0.63 ± 0.10  0.96 ± 0.03  0.43 ± 0.01 | 1.89 ± 0.35  0.28 ± 0.11  0.68 ± 0.16  1.08 ± 0.04  0.44 ± 0.02 | 1.38 ± 0.30  0.38 ± 0.14  0.69 ± 0.12  1.17 ± 0.04  0.44 ± 0.02 |

Values are the calculated average of two biological duplicates of growth of the indicated strains on 8% glucose and 4% xylose. The errors indicated are the standard error of the mean.

Q: specific productivity, Y: yield, P: productivity

**Supplementary Table 2.** **Primers used for chimeric HXT11/2**

| Name | Sequence (5’ 🡪 3’) |
| --- | --- |
| F HXT11  R HXT11  F HXT2  R HXT2  F HXT2 TM 1  R HXT2 TM 1 | GGCCTCTAGAATGTCAGGTGTTAATAATACATCCGC  AATTCCCGGGTCAGCTGGAAAAGAACCTCTTGTAAATTG  GGCCTCTAGAATGTCTGAATTCGCTACTAGCCGC  AATTCCCGGGTTATTCCTCGGAAACTCTTTTTTCTTTTGAG  GCCGCATATTGGACTGTTATCTGTTTATGT  ACATAAACAGATAACAGTCCAATATGCGGCGAGGGGTTTTTGAGGTAAGTCAATAGG |

**Supplementary Table 3.** **Primers used for saturation mutagenesis of chimeric HXT11/2**

| Name | Sequence (5’ 🡪 3’) |
| --- | --- |
| F HXT11  R HXT2  F HXT2 361NNN  R HXT2 361NNN  F HXT2 N361L  R HXT2 N361L  F HXT2 N361Y  R HXT2 N361Y  F HXT2 N361H  R HXT2 N361H  F HXT2 N361D  R HXT2 N361D  F HXT2 N361E  R HXT2 N361E  F HXT2 N361C  R HXT2 N361C  F HXT2 N361W  R HXT2 N361W  F HXT2 N361M  R HXT2 N361M | GGCCTCTAGAATGTCAGGTGTTAATAATACATCCGC  AATTCCCGGGTTATTCCTCGGAAACTCTTTTTTCTTTTGAG  CTTTCCAAACTTCCATCGTTTTAGGTATAGTCnnnTTCGCATCCACTTTCGTGGC  GCCACGAAAGTGGATGCGAAnnnGACTATACCTAAAACGATGGAAGTTTGGAAAG  CTTTCCAAACTTCCATCGTTTTAGGTATAGTCcttTTCGCATCCACTTTCGTGGC  GCCACGAAAGTGGATGCGAAaagGACTATACCTAAAACGATGGAAGTTTGGAAAG  CTTTCCAAACTTCCATCGTTTTAGGTATAGTCtatTTCGCATCCACTTTCGTGGC  GCCACGAAAGTGGATGCGAAataGACTATACCTAAAACGATGGAAGTTTGGAAAG  CTTTCCAAACTTCCATCGTTTTAGGTATAGTCcatTTCGCATCCACTTTCGTGGC  GCCACGAAAGTGGATGCGAAatgGACTATACCTAAAACGATGGAAGTTTGGAAAG  CTTTCCAAACTTCCATCGTTTTAGGTATAGTCgatTTCGCATCCACTTTCGTGGC  GCCACGAAAGTGGATGCGAAatcGACTATACCTAAAACGATGGAAGTTTGGAAAG  CTTTCCAAACTTCCATCGTTTTAGGTATAGTCgaaTTCGCATCCACTTTCGTGGC  GCCACGAAAGTGGATGCGAAttcGACTATACCTAAAACGATGGAAGTTTGGAAAG  CTTTCCAAACTTCCATCGTTTTAGGTATAGTCtgtTTCGCATCCACTTTCGTGGC  GCCACGAAAGTGGATGCGAAacaGACTATACCTAAAACGATGGAAGTTTGGAAAG  CTTTCCAAACTTCCATCGTTTTAGGTATAGTCtggTTCGCATCCACTTTCGTGGC  GCCACGAAAGTGGATGCGAAccaGACTATACCTAAAACGATGGAAGTTTGGAAAG  CTTTCCAAACTTCCATCGTTTTAGGTATAGTCatgTTCGCATCCACTTTCGTGGC  GCCACGAAAGTGGATGCGAAcatGACTATACCTAAAACGATGGAAGTTTGGAAAG |

n is any nucleotide
